# Typologies of Intimate Partner Violence Against Women in Five Latin-American Countries: A Latent Class Analysis

**DOI:** 10.3389/ijph.2022.1604000

**Published:** 2022-08-19

**Authors:** Alexandra Restrepo, Nilton Montoya, Laura Zuluaga

**Affiliations:** ^1^ Epidemiology Research Group, School of Public Health, University of Antioquia, Medellín, Colombia; ^2^ Statistical Applications and Public health Group, School of Public Health, University of Antioquia, Medellín, Colombia; ^3^ University of Antioquia, Medellín, Colombia

**Keywords:** Public health, intimate partner violence, family violence, female victimization, violence against women

## Abstract

**Objectives:** To estimate typologies of Intimate Partner Violence against women in some Latin-American countries.

**Methods:** Multistage sampling survey included women aged 15 to 49 (*n* = 63,321). Latent class analysis was estimated, including psychological, physical, and sexual violence and control.

**Results:** The three-class model had a better fit. 1) The high-level IPV class (23%) comprised those suffering high levels of violence. They had higher education and wealth index, lived in urban settings, and their husbands used alcohol more. 2) The middle-level IPV class (45%) suffered high levels of control but low levels of other violence. They justified IPV more than other classes and this group had a high proportion of women without education. 3) Women in the non-IPV class (32%) did not report IPV.

**Conclusion:** Three typologies of IPV were found: high-level, middle-level, and non-IPV. Policies should create screening, early prevention strategies, and programs based on these typologies. The high-level IPV group can benefit from intense legal and mental health interventions, including alcohol reduction and women’s empowerment. The middle-level IPV group could benefit from interventions to reduce violence justification and increase women’s education.

## Introduction

Intimate Partner Violence (IPV) has been defined as follows: “Intimate Partner Violence refers to any behavior within an intimate relationship that causes physical, psychological or sexual harm to those in the relationship” [1]. Traditionally, IPV has been classified by violence mechanisms: physical aggression, psychological abuse, forced intercourse, or dominance or control (monitoring the partner’s relationships or behaviors, or restricting access to information or assistance) [1]. Alternatively, victims and perpetrators of IPV can be grouped according to specific patterns of behavior, and the patterns may have specific associated factors. Some studies have identified typologies of IPV in high-income countries [2–4]. Even though IPV and its consequences are widespread in Latin American countries, we did not find studies that have explored typologies of IPV, and the factors associated with each typology.

The prevalence of intimate partner physical or sexual violence against women in the world varies widely [5, 6] A systematic review of IPV prevalence in the Americas found that 14–17% of women reported physical or sexual violence in Brazil, Panama, and Uruguay, while 60% have experienced this form of violence in Bolivia [7]. Domestic violence leads to nine million years of life lost (YLL) per year worldwide, more than the total loss caused by all types of cancers affecting women [8]. In 1999, the estimated costs of domestic violence in Chile and Nicaragua were $1.73 billion and $32.7 million, respectively [8]. In Colombia, the direct costs of IPV were 11.5% of the GDP, and the indirect costs were 8.9% of the GDP in 2008 alone [9].

In a study of women in shelters in the 1990s, Johnson postulated that within IPV there are different types of offenders and victims [2]. Through cluster analysis, Johnson found four types of IPV: 1) Coercive Controlling Violence [10], which combines physical violence and control or dominance (generally exercised by men), quickly escalates to more severe forms, and affects victims’ mental health [11, 12]; 2) Situational Intimate Partner Violence, in which both partners engage in violence but not continuously or severely; 3) Mutual Violence Control, in which both intimate partners are violent; and 4) Violent Resistance, in which one partner (usually a woman) physically defends herself against violence perpetrated by the other partner [11, 12].

Following Johnson’s findings, Graham-Keven and Archer published studies with similar findings [3, 13]. These studies have provided insight into the types of IPV reported by those who come to health and wellbeing services [14] and have facilitated the development of penalties and treatment programs for offenders.

However, studies in representative samples of general populations have found different categories and distributions of IPV. One study found that 89% of the violence was Situational Intimate Partner Violence, and 11% Coercive Controlling Violence [10]. Further, population surveys have found that IPV victims were not as predominantly female as in shelter and criminal justice samples [15], in which Situational Intimate Partner Violence accounted for 29% and Coercive Controlling Violence for 68%, and in which men are more frequently the perpetrators [16].

The different typologies of IPV, related factors, and partners may change across different cultures and over time. Understanding these patterns may enable us to generate more specific strategies and programs for treatment and prevention. In this study, we conduct a comparative analysis in Latin America, using Demographic and Health Surveys (DHS) from Colombia, Dominican Republic, Haiti, Honduras, and Peru. This paper proposes two aims:

1) To estimate typologies or profiles of IPV against women and differences in these classes by country. Typologies of IPV were estimated using Latent Class Analysis (LCA) and we performed a multigroup latent class analysis to estimate differences between countries in the classes.
2) To describe each typology according to demographic characteristics of the women that belong to each class.

## Methods

We analyzed the DHS databases of Colombia (2015), the Dominican Republic (2013) [17], Haiti (2016–2017), Honduras (2011–2012), and Peru (2014). These surveys were conducted by governmental institutions in each country and Macro Inc.

The DHS surveys were probabilistic and involved multistage interviews of women aged 15–49 years. Data were collected in rural and urban areas, and the surveys were designed to support inferences within countries and regions [18]. In each country, the investigators selected a subsample of women who answered the questions about domestic violence and conducted interviews privately (without family members present) because the questions were sensitive. (For more details about methodology and sampling, see http://dhsprogram.com/data/).

We chose these countries’ surveys because they were household surveys involving representative samples, used similar questionnaires, and methods, had temporal proximity, and had question uniformity. Supplementary Table S1 includes a description of each country’s survey methods.

### Sample

The original sample was 109,022 women between 15 and 49 years old. We selected women from each study who reported having been married, completed the intimate partner violence questionnaire, and were interviewed in a private setting without the interference of other family members or their husbands. The final sample for the analyses included 63,321 women (Final samples by country: Colombia = 24,890, Dominican Republic = 5,801, Haiti = 6,650, Honduras = 12,497, and Peru = 13,485).

### Outcome Variables

#### Violence Against Women From the Intimate Partner in Their Lifetime

The survey asked women if they were victims of different forms of IPV at any point in their lifetime. The responses were coded as 0 = they were never victims and 1 = they were victims of at least one form of violence. We calculated a variable for each type of IPV. 1) Control in life included the following situations: husband accuses her of unfaithfulness, the husband tries to limit her contact with family, the husband does not permit her to meet her friends, the husband insists on knowing where she is, and the husband does not trust her with money. 2) Physical Violence included slapping and threatening with a knife or another weapon, pushing or pinching with something harmful and attempting to strangle or burn, physical violence with injury (bruises, wounds, or broken bones). 3) Sexual violence: Intimate partner tried to force sex upon her*.*

A variable related to violence by women against their intimate partners was included: Ever physically hurt by the husband when the woman was not hurting him. We coded this variable as a dichotomous variable (1 = Yes, and 0 = No).

### Demographic Variables

**•** Sociodemographic characteristics of women: 1) Woman’s age in years. 2) Woman’s highest educational level was measured in four categories (No education = 0, Basic school = 1, High school = 2, and University studies = 3) 2) Women’s working status (1 = Yes, and 0 = No).
• Family and house characteristics. 1) Place of residence (Urban = 1 and Rural = 0), 2) Number of household members, 3) The number of children five-years-old and under, 4) Wealth index: This variable was calculated through a Principal Components Analysis by the DHS and measures the poverty level. Wealth index was calculated using variables, such as ownership of goods, including radios or automobiles, and house characteristics (i.e., type of floor and roof, bathrooms, and water). This variable was divided into quintiles: poorest, poor, middle class, rich, and richest [19, 20].
• Intimate partner variables: Some characteristics of the intimate partner were included, such as: 1) Intimate partner’s age in years at the time of the survey. 2) Intimate partner’s educational attainment measured in four categories: Does not have education = 0, Basic school = 1, High school = 2, and University studies = 3.

### Sample Description

For the overall sample, the average female age was 33.1 years (33.09–33.14), and these means were similar among countries. Concerning the educational level, a higher proportion of women who finished high school were found in Colombia (Proportion = P [95% CI] = 45.7% [45.1–46.3]). The highest proportion of women with university studies was found in Colombia (P [95% CI] = 27.1% [ 26.5–27.6]) and the lowest proportion of women who finished university studies were in Haiti (P [95% CI] = 3.5% [3.1–4.0]) and Honduras (P [95% CI] = 5.0% [4.6–5.4]) (Table 1).

**TABLE 1:** Demographic characteristics of the sample of the Demographic and Health Surveys of Colombia (2015), Dominican Republic (2013), Haiti (2016–17), Honduras (2011–12), and Peru (2014). Typologies of Intimate Partner Violence in Latin-American countries. 2013–2017.

Characteristic	Total	Country				
		Colombia	Dominican Republic	Haiti	Honduras	Peru
		(*n* = 24,890)	(*n* = 5,795)	(*n* = 6,627)	(*n* = 12,480)	(*n* = 13,483)
Woman’s current age (mean and 95% CI)	33.1	33.9	32.3	32.5	31.3	34
	(33.09–33.14)	(33.86–33.93)	(32.27–32.43)	(32.41–32.55)	(31.29–31.39)	(33.92–34.01)
Highest educational level						
No education	5.9	2.6	3.6	24.8	5.9	3.8
	(5.7–6.1)	(2.4–2.8)	(3.1–4.1)	(23.8–25.9)	(5.5–6.3)	(3.5–4.1)
Basic school	37.1	24.7	37.5	41.4	63.5	33.4
	(36.7–37.5)	(24.1–25.2)	(36.2–38.7)	(40.2–42.6)	(62.6–64.3)	(32.6–34.2)
High school	37.9	45.7	34.8	30.2	25.7	39.9
	(37.5–38.3)	(45.1–46.3)	(33.6–36.0)	(29.1–31.4)	(24.9–26.5)	(39.1–40.7)
University	19.1	27.1	24.2	3.5	50	22.9
	(18.8–19.4)	(26.5–27.6)	(23.1–25.3)	(3.1–4.0)	(4.6–5.4)	(22.2–23.7)
Respondent currently working						
No	43.6	39.8	50.4	45.9	57.8	33.4
	(43.2–44.0)	(39.2–40.4)	(49.1–51.7)	(44.7–47.1)	(56.9–58.7)	(32.6–34.2)
Yes	56.4	60.2	49.6	54.1	42.2	66.6
	(56.0–56.8)	(59.6–60.8)	(48.3–50.9)	(52.9–55.3)	(41.3–43.1)	(65.8–67.4)
Type of place of residence						
Urban	60.2	72.9	70.7	40.4	38.5	61.9
	(59.8–60.6)	(72.4–73.5)	(69.5–71.8)	(39.2–41.6)	(37.7–39.4)	(61.0–62.7)
Rural	39.9	27.1	29.4	59.6	61.6	38.1
	(39.5–40.2)	(26.5–27.6)	(28.2–30.6)	(58.4–60.8)	(60.7–62.4)	(37.3–39.0)
Wealth index						
Poorest	24.5	27	24.9	22.3	25.5	20.1
	(24.2–24.8)	(26.5–27.6)	(23.8–26.0)	(21.3–23.3)	(24.7–26.3)	(19.5–20.8)
Poorer	25.5	30.4	23.1	19.8	22.9	23
	(25.2–25.9)	(29.9–31.0)	(22.0–24.2)	(18.8–20.8)	(22.2–23.7)	(22.3–23.7)
Middle	21.3	20.8	20.1	24.7	20	22.5
	(21.0–21.7)	(20.3–21.3)	(19.0–21.1)	(23.7–25.8)	(19.3–20.7)	(21.8–23.1)
Richer	16.6	13.5	17.8	18.8	17.6	19.6
	(16.3–16.9)	(13.0–13.9)	(16.8–18.8)	(17.9–19.8)	(17.0–18.3)	(18.9–20.2)
Richest	12	8.3	14.2	14.3	14	14.8
	(11.8–12.3)	(8.0–8.7)	(13.3–15.1)	(13.5–15.2)	(13.4–14.6)	(14.3–15.4)
Husband/partner’s age (mean and CI)	37.4	38.3	37.7	38.1	35.7	37.7
	(37.42–37.47)	(38.29–38.38)	(37.6–37.77)	(38. - 38.16)	(35.61–35.72)	(37.68–37.78)
Husband/partner’s educational attainment						
No education	4.4	0.6	4.4	18.7	7.7	1.4
	(4.3–4.6)	(0.5–0.7)	(3.9–5.0)	(17.8–19.7)	(7.2–8.2)	(1.2–1.6)
Basic school	27.8	5	41	35.8	65.4	25.2
	(27.4–28.1)	(4.7–5.3)	(39.7–42.3)	(34.7–37.0)	(64.6–66.3)	(24.5–26.0)
High school	13.2	6.2	16.3	3.6	8.2	33.9
	(12.9–13.4)	(5.9–6.5)	(15.4–17.3)	(3.1–4.0)	(7.7–8.6)	(33.1–34.7)
University	20.2	2.9	32.9	38.6	18.6	39
	(19.9–20.5)	(2.7–3.1)	(31.7–34.1)	(37.4–39.7)	(17.9–19.2)	(38.2–39.9)

The proportion of poorest population was greater in Colombia (P [95% CI] = 27.0% [26.5–27.6]), and Honduras (P [95% CI] = 25.5% [24.7–26.3]) compared with the other countries. The place of residence was more frequently urban in Colombia (P [95% CI] = 72.9% [72.4–73.5) and in the Dominican Republic (P [95% CI] = 70.7% [69.5–71.8]), while the highest proportion of the population living in rural areas (P [95% CI] = 61.6% [60.7–62.4]) was found in Honduras (Table 1).

Concerning husband characteristics, the mean age was 37.4 (95% CI: 37.2–37.40) years, and this average was similar between the three countries (Table 1).

### Statistical Analyses

• Sample Description: We describe the sample according to demographic characteristics. Even though previous reports had described the DHS sample characteristics, we presented a descriptive analysis of the sample used for the current article because the analysis was performed in a subsamples (i.e., women between 15- to 49-years-old, married, and who completed the IPV questionnaire). We calculated proportions using percentages and 95% confidence intervals (CI). For women and husband/partner age, we used the mean and 95% CI.
• Construction of the Latent classes: We used a LCA to estimate the typologies or profiles of women that suffered IPV. Using LCA, we classified study participants according to their verbal reports to create profiles of patients according to the severity of their symptoms [21]. In addition, LCA has been applied to classify the typologies of violence against adolescents according to severity [22]. Previous studies assessing typologies of IPV used the cluster analysis [2] technique, which also allowed for the classification of individuals but did not evaluate how this classification may vary according to country. Multigroup LCA allows us to detect if classes vary across the country because we considered the potential for sampling and cultural differences to influence the report of IPV. In this analysis, we had four forms of female victimization according to the aggression mechanism (i.e., control, physical aggression, severe physical aggression, and sexual aggression) and one form of female aggression against her partner. We calculated sequential latent class models in the overall sample using one to four classes. The candidate model was determined using BIC (Bayesian information criterion), loglikelihood criteria, AIC (Akaike information criterion), adjusted AIC (Adjusted Akaike information criterion), and a theoretical concordance.
  Latent class analysis by country: We conducted a multigroup LCA using the country as a group variable and testing the 3-class model. To proceed with the multi-group LCA, we calculated separate models for each country using the multigroup feature of Mplus and assessed differences among countries’ models.
  Description of latent classes: Participants were grouped according to the calculated probability of belonging to each of the three classes. We described each class based on demographics, women’s attitudes and beliefs, intimate partner characteristics, and family variables. We described categorical variables using proportions and CI. For women and husbands’ ages, we used the mean and CI. We used a chi-squared test to measure differences among proportions and ANOVA to test differences of means among classes.

## Results

### Latent Class Analysis

#### Latent Classes Estimation

We chose the 3-class model because it had better fit indexes and was more parsimonious and interpretable than the 2- and 4-class models. We named class 1 “high-level of IPV” because this class has high levels of all forms of aggression except for sexual aggression. This class represents 22.9% of the women in the sample. Eighty-eight percent of women who belong to the high-level of IPV were victims of control, 27.2% of sexual aggression, and nearly 100% were victims of physical aggression.

We named the second class “middle-level of IPV,” as this class has high levels of control (78%) but low levels of physical violence (7.7%), sexual violence (2%), and moderate levels of women’s aggression against partners (13%). This class represents 45.1% of the sample.

**FIGURE 1:**
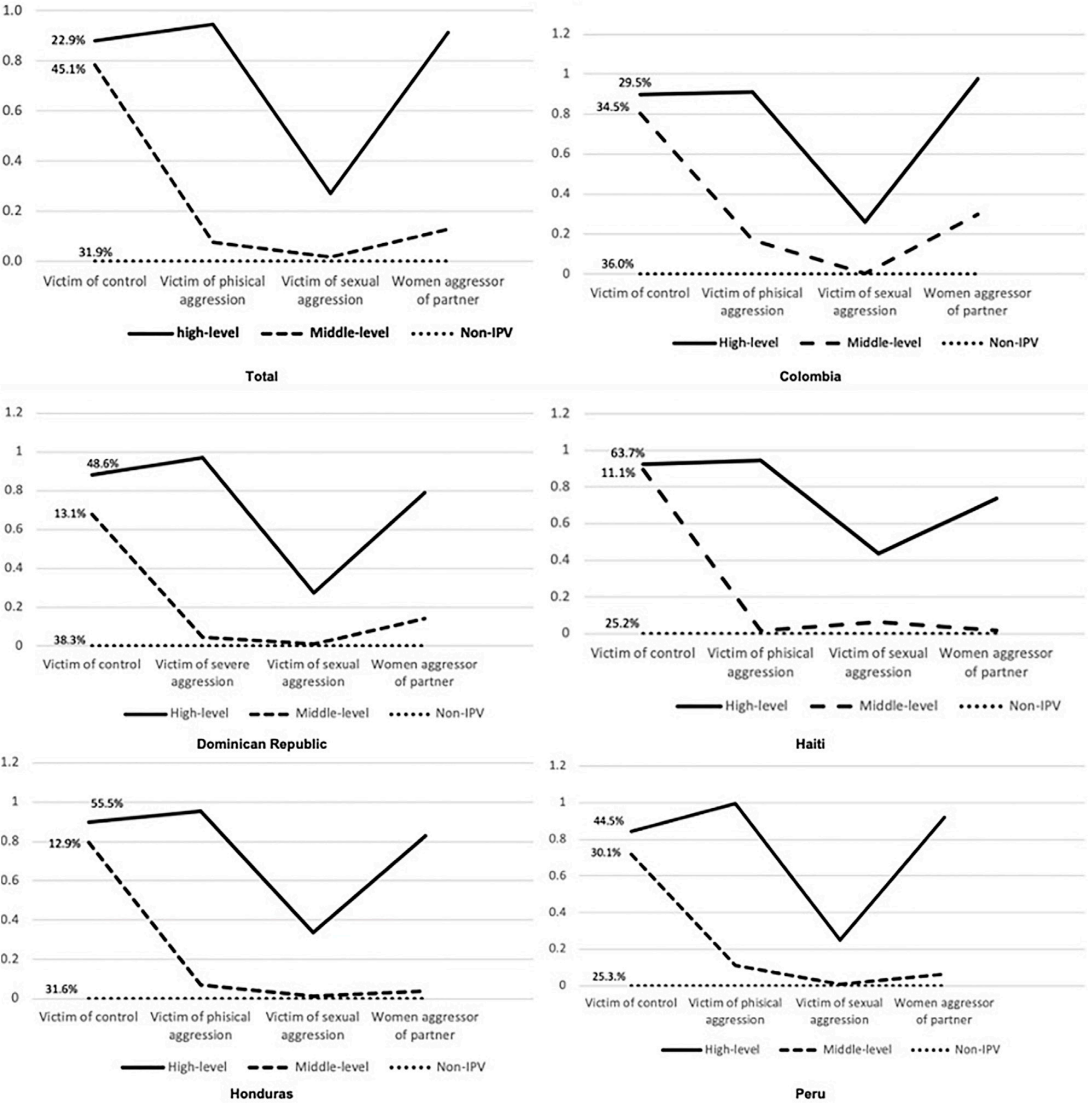
Latent Classes Analysis by country. Colombia (2015), Dominican Republic (2013), Haiti (2016–17), Honduras (2011–12), and Peru (2014). A Latent Class Analysis. Black line = high level of violence class, dashed line = middle-level IPV, and dotted line = non-IPV. Typologies of Intimate Partner Violence in Latin-American countries. 2013–2017.

Finally, we named class 3 “Non-IPV” because they did not suffer any form of violence (Model 3 in Tables 2, 3, and Figure 1). This Non-IPV class represents 31.9% of the sample (Model 3, Table 3).

**TABLE 2:** Latent Class Analysis and goodness of fit indicators for typologies of IPV. Colombia (2015), Dominican Republic (2013), Haiti (2016–17), Honduras (2011–12), and Peru (2014). Typologies of Intimate Partner Violence in Latin-American countries. 2013–2017.

Model	Loglikelihood	df	AIC	BIC	Adjusted BIC	Entropy	Absolute frequency for smallest class	Relative frequency for smallest class	Parametric bootstrapped likelihood ratio test
1-Class	−138018.782	4	276045.564	276082.45	276069.74	—	—	—	—
Model 1									
2-Class	−116396.342	9	232810.683	232893.676	232865.07	0.787	22299.51	29.85	*p* value = 0.000
Model 2									
3-Class	−115816.549	14	231661.098	231790.198	231745.71	0.685	15622.18	20.91	*p* value = 0.000
Model 3									
4-Class	−115691.82	19	231421.64	231596.847	231536.47	0.603	8308.20	11.12	*p* value = 0.000
Model 4									

df, degree of freedom; AIC, akaike information criterion; adjusted AIC, adjusted akaike information criterion; BIC, Bayesian information criterion.

**TABLE 3:** Descriptive latent classes according to the violence type. Colombia (2015), Dominican Republic (2013), Haiti (2016–17), Honduras (2011–12), and Peru (2014). Typologies of Intimate Partner Violence in Latin-American countries. 2013–2017.

				Chosen model								
	1-class	2-class model		3-class model			4-class model			
	Model 1	Model 2		Model 3			Model 4			
Forms of violence		Class 1	Class 2	Class 1	Class 2	Class 3	Class 1	Class 2	Class 3	Class 4	
n	63,306	16,851	46,470	14,512	39,982	20,219	8,308	17,379	17,311	31,716
Percentage in the population	100	26.6	73.4	19.4	53.5	27.1	11.12	23.26	23.17	42.45
Victim of control (%)	62.6	48.9	87.6	88.0	0.784	0.001	93.6	0.999	76.2	0.554
Victim physical aggression (%)	23.9	1.4	74.9	94.4	0.077	0.000	93.8	0.0	32.0	0.000
Victim of sexual aggression (%)	7.6	0.0	21.7	27.2	0.018	0.000	38.7	0.6	0.6	0.001
Woman aggressor of her partner (%)	28.9	2.9	78.7	91.2	0.128	0.000	89.8	19.1	61.3	0.043

#### Latent Class Analysis by Country

In Figure 1, we can observe the multigroup LCA by country (Likelihood = −200,773.135, AIC = 401,656.271, BIC = 402,154.336, BIC adjusted = 401,979.545). Therefore, we preferred the overall 3-class model to explain the class in the population. The probability of having each form of aggression given the latent class is similar between countries, with only minor differences. The proportion of those in the high-level IPV class ranged from 64% in Haiti to 29.5% in Colombia. Similarly, middle level-IPV ranged from 11% in Haiti to 30% in Peru. The Dominican Republic and Colombia had the highest percentage of participants in the non-IPV class (Figure 1).

#### Description of IPV Typologies

Women who belong to the high IPV class were 34 years old and had secondary (42%) or higher education (18.8%). Seventy-eight percent of the women in high-level IPV were working. The husband/partner was, on average, 42 years old, had primary (38%) or higher education (32%), and 74% of their husbands or partners drank alcohol. Finally, seven percent of the women in the high-level IPV class justified IPV, and 7.5% reported their partner made more than four important decisions alone (Table 4).

**TABLE 4:** Descriptive analysis of IPV classes according to women and husband/partner characteristics. Typologies of Intimate Partner Violence in Latin-American countries. 2013–2017.

Characteristic	IPV classes		
	High-level class	Middle-level	Non-IPV
Woman age in years (mean and 95% CI)	34.1 (34.0–34.3)	32.2 (32.1–32.3)	33.7 (33.6–33.9) ***
Woman education level			
No education	4.7 (4.4–5.1)	6.2 (5.9–6.4)	6.4 (6.1–6.8) ***
Primary	34.5 (33.8–35.3)	39.5 (38.9–40.0)	35.7 (35.0–36.3) ***
Secondary	42.0 (41.2–42.8)	37.1 (36.5–37.6)	36.1 (35.4–36.8) ***
Higher	18.8 (18.1–19.4)	17.3 (16.9–17.8)	21.8 (21.2–22.4) ***
Woman currently pregnant			
No or unsure	93.0 (91.7–94.3)	91.1 (90.4–91.7)	92.7 (91.9–93.5) **
Yes	7.0 (5.7–8.3)	8.9 (8.3–9.6)	7.3 (6.5–8.1) **
Woman worked past year			
No	21.8 (21.2–22.5)	34.0 (33.4–34.5)	36.2 (35.5–36.8)
Yes	78.2 (77.5–78.8)	66.0 (65.5–66.6)	63.8 (63.2–64.5)
Missing			
Partner/husband age in years (Mean and 95% CI)			
	41.6 (41.4–41.9)	41.4 (41.2–41.6)	41.8 (41.6–42.0) ***
Husband/partner education level			
No education	5.0 (4.5–5.5)	7.3 (6.9–7.6)	7.0 (6.5–7.4) ***
Primary	38.0 (36.9–39.0)	43.9 (43.2–44.6)	42.6 (41.8–43.5) ***
Secondary	25.5 (24.5–26.4)	17.9 (17.4–18.4)	20.3 (19.6–21.0) ***
Higher	31.6 (30.6–32.6)	30.9 (30.3–31.5)	30.1 (29.3–30.9)
Husband/partner drinks alcohol			
No	26.2 (25.2–27.2)	47.4 (46.7–48.1)	55.4 (54.5–56.3) ***
Yes	73.8 (72.8–74.8)	52.6 (51.9–53.3)	44.6 (43.7–45.5) ***
Wealthy index			
Poorest	24.5 (23.8–25.2)	24.5 (23.9–25.0)	25.8 (25.2–26.5) ***
Poorer	28.8 (28.0–29.5)	25.2 (24.7–25.7)	24.9 (24.3–25.5)
Middle	22.2 (21.5–22.9)	21.5 (21.0–22.0)	19.9 (19.3–20.5)
Richer	15.4 (14.8–16.1)	16.8 (16.4–17.3)	16.3 (15.8–16.9)
Richest	9.1 (8.6–9.6)	12.0 (11.6–12.4)	13.0 (12.5–13.5)
Place of residence			
Urban	67.0 (66.2–67.7)	57.2 (56.7–57.8)	59.4 (58.7–60.1)
Rural	33.0 (32.3–33.8)	42.8 (42.2–43.3)	40.6 (39.9–41.3)
Woman justify IPV			
Yes	7.2 (6.8–7.6)	9.0 (8.7–9.4)	4.9 (4.6–5.2)
No	92.8 (92.4–93.2)	91.0 (90.6–91.3)	95.1 (94.8–95.4)
Number of household decisions made only by the husband			
0 decisions	63.6 (62.8–64.4)	59.4 (58.8–59.9)	64.8 (64.2–65.5)
1–3 decisions	28.9 (28.2–29.7)	33.2 (32.7–33.8)	28.8 (28.2–29.4)
More than 4 decisions	7.5 (7.1–7.9)	7.4 (7.1–7.7)	6.4 (6.0–6.7)

*<0.05, ** <0.01 *** < 0.001.

a Chi-squared test difference among n proportions.

b ANOVA, models.

Regarding women in the middle-level IPV class, we found they were, on average, 32 years old, and most of them had completed primary (40%) or secondary education (37%). Sixty-six percent had been working in the past year. The mean age of their partners was 43 years old, and their partners had secondary education (44%). Regarding the level of wealth, close to 50% were in the poorer and poorest wealth levels (Table 4).

Finally, women belonging to the non-IPV class were, on average, 33.7-years-old, and most of them had primary (36%) or secondary education (36%). Six percent of women in the non-IPV class reported that their partner made more than four important decisions at home alone, and 5% justified domestic violence (Table 4).

When comparing classes, we found that women and their partners had similar ages. Women in the high-level IPV class had a higher education level than women in the middle- and non-IPV classes. Sixty-one percent of women in the high-level IPV class had secondary education or more, while 52% of women in the middle and non-IPV class had more than secondary education. In addition, a high proportion of women in the high-level IPV class (67%) lived in urban areas compared with the other classes (58% in middle-level IPV and 59% in non-IPV class). There were no differences among classes according to the wealth index (Table 4).

Women in the middle-level and non-IPV classes had a higher proportion of no education than the high-level class. Similarly, their partners/husbands had a higher proportion of no education than the high-level class.

Further, women in the high-level (7.5%) and middle-level (7%) classes reported that a higher proportion of their husbands/partners make decisions alone at home compared with the non-IPV class (6%). In addition, women in the middle-class justified domestic violence more than women in other classes (Table 4).

Finally, regarding their husbands/partners, women in the high-IPV class had husbands/partners who had a higher proportion of alcohol use than middle- and non-IPV classes.

## Discussion

### Estimates and Description of IPV Typologies

To assess the typologies of IPV, we conducted an LCA exploring models ranging from 1 to 4 classes. We chose the model with three latent classes because this model was parsimonious and had a better fit. We named the three classes according to the level of violence as high-level, middle-level, and non-IPV classes.

Women in the high-level IPV class were victims of high levels of control, physical violence, and were aggressors of their partners/husbands.

Women in the high-level IPV class had a higher education than women in other classes; their husbands used more alcohol and predominantly lived in urban settings. Women in the middle-level of IPV class suffered high levels of control but low levels of physical and sexual violence. Women in the middle-level and high-level IPV classes justified IPV more, and there was a higher proportion of women without education than in the high-level IPV class. Women and their husbands/partners from the middle- and non-IPV classes had a higher proportion of no education. Finally, participants in the non-IPV class had not been victims or aggressors of IPV forms.

The typologies we obtained in our analysis differed from those described by Johnson [11] and Strauss [15]. The high-level categories could be similar to the mutual violence control type described by Johnson [11] because this class also had high levels of all forms of intimate partner violence. The non-IPV class was similar to the cluster of situational violence described by Johnson as the lower level of violence. However, in our model, women were not aggressors of their partners. These results were congruent with those shown in three systematic reviews about variables associated with IPV [23–25].

This paper is the first study to address IPV typologies in Latin-American and low-middle income countries, settings with scarce prior evidence and datasets that previously lacked research on this topic. This research provides information about different countries and describes the different identified classes according to demographic and social characteristics. We also included different forms of victimization as well as female aggression to the partner/husband, providing a more compressive depiction of the violence dynamics in partnerships.

### Limitations

Among the limitations of this study, we discuss the bias due to differences in the sampling, bias because of the differences in study focus, misclassification of the outcome, women underreporting domestic violence, and cultural differences regarding domestic violence among countries.

First, we consider that bias resulting from sampling differences are minimal because these surveys were all collected based on multistage sampling procedures performed similarly between countries, since Macro International uses similar sampling methodologies and women selection to guarantee comparability between countries. In addition, surveys were collected in different years; however, their intimate partner violence is not a seasonal event.Instead, IPV is a health outcome explained by structural risk factors. Also, the surveys included the same target population allowing comparisons of countries. The target populations are women aged 12–49 in all the surveys from all education and socioeconomic levels across countries.

Second, bias due to differences in the study aims and survey focus is minimal. The focus of the surveys was similar among countries; they aimed to estimate demographic indicators, women’s health behavior, intimate partner violence, pregnancy, immunizations, and practices in Sexual and Reproductive Health (Supplementary Table S1). Specifically, regarding the aim of IPV, we found that all countries’ surveys sought to describe the proportion of women victims of IPV.

The third limitation is the possible misclassification of the outcome. We considered that a misclassification bias of IPV could arise because questionnaires are not validated. However, the questionaries about IPV are similar among countries. The questions regarding IPV and demographic variables used in this analysis were comparable between counties. Macro international used the same questionaries and methodologies across countries to maintain comparability. For this reason, there are publications including multiple countries using this data [26]. The team also harmonized the questionaries by reviewing each question-and-answer category.

The fourth limitation is the potential for information bias that could arise from women not reporting their actual exposure to IPV due to fears surrounding reporting aggression, which would result in underestimating the proportion of domestic violence and could affect the identification of IPV typologies.

Finally, the cultural and social differences among the counties studied could affect IPV reporting due to more permissive attitudes or cultural acceptance of different forms of violence. Information bias can arise because the participants could be more reluctant to report being victims of domestic violence in those countries where IPV was more accepted or where women are less empowered about their rights. The underreporting of domestic violence could generate a different distribution of latent classes. Honduras has a marked patriarchal culture that increases the likelihood of indigenous populations underreporting domestic violence [27]. The Dominican Republic is a Caribbean country where gender inequities persist, as do rigid gender roles and a high level of social acceptance of IPV [28]. In Colombia, the gender power imbalance driven by social inequalities, low empowerment of women’s rights, and low education level persists [29, 30]. In Peru, low education levels, unemployment, and a history of family violence are important risk factors for IPV victims [31]. Peru has a high proportion of women from ethnic minorities, with higher levels of violence and more economic and social disparities [32]. Haiti had high levels of poverty and violence. In addition, social norms maintain gender imbalances, women’s poverty, and acceptance of IPV, leading to reduced reporting of IPV [33, 34]. For all these differences among countries, we conducted an overall latent class model and tested the multigroup latent class in which we found classes were similar between countries.

### Recommendations for Policies and Public Health Programs

Policies and services should be prepared to identify and treat couples according to their typology, implement screening tests to classify couples according to these typologies, and perform interventions according to their type. First, policies should screen women in health and well-being services to determine which type of IPV class they belong to. Second, policies should include not only strategies to punish the IPV aggressors but also promote health and early prevention strategies. For instance, we need more strategies to empower women and shift those cultural beliefs that IPV is acceptable, thus hampering the vicious cycle that perpetuates domestic violence through generations. Also, policies should include health promotion programs that reduce the socioeconomic gaps by providing equal opportunities in employment and education for both genders. Third, policies should create and provide funding for differential interventions that consider the IVP typologies. Women in the high-level IPV class should be included in integrative interventions that remove women and their children from the violent environment and provide free access to legal and mental health services. Policies for the middle-level should include education for women and their husbands, conflict resolution, and women’s empowerment.

Women with middle-levels of IPV could benefit from an intervention that addresses IPV justification and increases women’s education level. Those couples with high levels of IPV should benefit more from extensive interventions that include counseling, legal services, and alcohol reduction. As women and their husbands/partners in this class had high education levels and were wealthy, women may benefit from interventions to empower their rights and conflict resolution. Women with high levels of IPV may also need mental health and stress management interventions to address the possible consequences of this intervention’s mental health. Finally, both high and middle-level IPW could benefit from interventions that improve communication, conflict resolution, and stress management.

### Conclusion

We found three typologies of IPV: high-level, middle-level, and non-IPV. Policymakers should create programs to prevent and treat different forms of violence rather than treating domestic violence as a uniform phenomenon. First, according to these typologies, policies should create screening methods that detect and intervene in the population. Second, policies should include strategies to punish IPV aggressors and promote health and early prevention strategies that reduce IPV approval, increase women’s empowerment about their rights, and increase their socioeconomic and education opportunities. Police should intervene according to IPV levels. Women who had high levels of IPV could benefit from programs that include legal and mental health services, alcohol reduction, cultural change, and women’s rights empowerment. Women who are victims of middle levels of IPV could benefit from interventions to reduce the justification of IPV and increase women’s education levels. Women with middle- and high-IPV may need interventions to increase conflict resolution skills and stress management.

## Abbreviations

CI, confidence Interval; DHS, demographic Health Survey; IPV, intimate partner violence; LCA, latent class analysis; WHO, World Health Organization; WI, wealth Index.
