# Supplementary material for: Typologies of Intimate Partner Violence Against Women in Five Latin-American Countries: A Latent Class Analysis
Source: Int J Public Health. 2022 Aug 19;67:1604000. doi: 10.3389/ijph.2022.1604000 (PMC9437212; doi:10.3389/ijph.2022.1604000)
Supplement: Supplementary file 1 [file Table1.docx]

**Supplement table 1. Characteristic of Demography and Health Surveys of Colombia (2015), the Dominican Republic (2013), Haiti (2016-2017), Honduras (2011-2012), and Peru (2014).**

| **Characteristic/sample** | **Colombia** | **Dominican Republic** | **Haiti** | **Honduras** | **Peru** |
| --- | --- | --- | --- | --- | --- |
| **Year** | 2015 | 2013 | 2016-2017 | 2011-2012 | 2014 |
| **Aim** | To establish the  demographic changes of the Colombian population occurred  in the last 5 years (2010-2015).  obtain information  about knowledge, attitudes, and practices in  Sexual Health and Reproductive Health of women and men in  fertile age. | To obtain reliable estimates on several indicators such as:   - Fertility - Health status - Reproductive health - Nutritional status of children - child mortality - HIV / AIDS - Intrafamily violence, - Morbidity - Other: tuberculosis, smoking, diet and exercise, and preventive health care | To updated indicator estimates   - Demographic and basic health. - Child and maternal health, - family planning, nutrition - health behavior and knowledge - health care access and use - immunization. - Domestic violence - Other: HIV, Diabetes Mellitus, and hypertension | To provide updated statistical information at the national level and by departmental and metropolitan area including   - Fertility - Health status - Reproductive health - Nutritional status of children - child mortality - HIV / AIDS - Intrafamily violence, - morbidity - Utilization of services - Demographic indicator | To provide information about most important demographic and health indicators such as:   - Reproductive health - Maternal and child health - Contraceptive use methods, - Care of pregnancy and delivery - Immunizations. - Prevalence diseases among child population - Nutrition - AIDS and domestic violence. |
| **Population included** | Women children under 5 years, and man | Women, children under 5 years, and man | Women, children under 5 years, and man | Women, children under 5 years, and man | Women and children under 5 years old |
| **Ages included** | Women aged 13-49 and men aged 13 -59 | Women aged 15–49 and men aged 15-64 | Women aged 15-49 and men aged 15-64 | Women aged 15 to 49 and men aged 15-59 | Women aged 15-49 |
| **Households sample size** | 44,614 | 11,464 | 13,405 | 21,362 | 28 185 |
| **Individuals sample size** | Women= 38,718 and Men=35,783 | Women=9,372 and men = 10,306 | Women= 14,371 and men= 9,795 | Women=22,757 men=7120 | Women =24,872 |
| **Subsample included in analysis** | 24,890 | 5,801 | 6,650 | 12,497 | 13,485 |
| **Sampling methods** | Probabilistic, cluster, stratified and multistage | Probabilistic, cluster and two-stage sampling | Probabilistic, cluster and two-stage sampling | probabilistic at the national level, which is stratified and two-stage. | Probabilistic, cluster and two-stage sampling |
| **Response rate** | 87.3% | 90.3 | 98.5 | 89.6 | No reported |
| **Inference level** | Country.  Region and subregions  States  Rural/urban | Country  Region  States  Rural/urban | Country  Regions  States  Rural/urban | Country  States  Rural/urban | Country  States  Rural/urban |

This table summarizes the characteristics obtained from countries' reports. DHS. Publications by Country. Rockville: DHS (2008). Available from: http://

www.dhsprogram.com/publications/Publications-by-Country.cfm
